# Editorial

**Published:** 1857-09

**Authors:** 


### Editorial.

### THE AMERICAN DENTAL CONVENTION.

The Third Annual National Convention of Dentists, was held at Boston on the 4th, 5th, 6th, and 7th of August.

To those who were there, anything we might say here would only mar the pleasant recollection of the good time they enjoyed, and to those who were not there, it would be difficult to give anything like an accurate idea of what was done.

The Convention met at 12 o'clock M., on Tuesday, and proceeded at once to the election of officers, which was soon done,—with rather more dispatch, than heretofore.

The topics presented by the executive committee for discussion were good, and of much importance to the dentist.

These points were taken up and pretty thoroughly discussed, at least so far as time was concerned. Many of the members engaged in the discussions with considerable zeal and at great length.

The saleratus question was put through by day-light. It knocks the "*goose question*" into fits.

The discussions in the main were very interesting, especially the descriptions of various opeiations and processes. Much, no doubt, was learned by all, from these discussions and descriptions.

The time was limited to ten minutes, and many were thus cut off from giving full descriptions. We do think, that in these meetings ample time should be given to every one who has anything *new or interesting* to bring it forward. And in order to do this the session should continue at least one week.

We would ventuie to suggest that it is not best to have protracted repetition, either by one or a number of persons. It would probably be better if the spirit would not move too many men to speak at the same time,—move one at a time and move him *effectually*.

That trip down the bay on Thursday afternoon. Oh, what a time! The Boston dentists will long be remembered for it. Scarcely, in the absence of ladies, did any crowd ever enjoy themselves so well. The good boat, a fine captain—the pleasant sailing—the ample provision for the inner-man, in the way of refreshments, and—and—and *fishing* and Nahant *of sea snaix* notoriety, and getting stuck fast in the *mud*, and Hull, and the good things Dr. Tucker had and made common, and the glorious music, it was the finest that is made. The sailing round the bay by moonlight, entranced by sweet sounds, etc., etc.

To the Boston dentists thanks and honor, for the numerous exhibitions of kindness and generosity manifested to those of us who were strangers and sojourners there.

The Convention met on Friday morning, and after some discussion on practice; and another matter which we notice elsewhere,—adjourned, and will meet at Cincinnati on the first Tuesday of August, 1858. And we hope every man will come prepared to keep the ball in motion till Saturday evening.

---

### FUND FOR THE PROMOTION OF DENTAL SCIENCE.

The American Dental Convention, which met at Boston, in August last, considered and passed the following resolutions:

*Resolved,* That this Convention establish a fund for for the promotion of Dental Science, with especial reference to the employment of some competent person or persons to conduct experiments—physiological, pathological, chemical and hygienic, as connected with dental science,

2d. *Resolved,* That a supervisory committee of three from the dental profession in the United States be appointed whose duty it shall be to take charge of the whole matter. That committee shall have its chairman, secretary and treasurer. It shall also be the duty of that committee to secure the services immediately, of some competent person or persons, as experimenter, who shall as soon as possible begin his labor. And that the results of such labors shall be given to the profession as soon as they are obtained.

3d. *Resolved,* That such person be placed in the most favorable position by the profession of the United States for carrying out the objects of the first resolution.

Every one will perceive at a glance, the objects aimed at in the above resolutions. It is to open up the hidden treasures of the collateral sciences, and make them tributary, more than hitherto, to the dental specialty, and to bring to full view, things that have as yet, been seen only in the dim and uncertain twilight, as well as to bring forth, from total obscurity, agents, processes and principles, that shall develop our young science with far greater rapidity and certainty than heretofore.

There are many things in the practice of our profession involved in uncertainty and obscurity. Many have been the efforts of practitioners, to execute experiments for the purpose of bringing out and demonstrating, the truth or fallacy of something about which they have long been in doubt. And yet how few succeed in accomplishing anything, in that way. And it would be wonderful indeed if much should be accomplished. I suppose it to be a safe estimate, that nineteen twentieths of all the experiments thought of and attempted by dentists, prove total failures, so far as the desired result is concerned. Under the circumstances it could not be otherwise. I do not think it is disrespectful, nor slandering the profession, to say that the great majority of them are not competent just at once and without considerable preparation to go to work, and efficiently, introduce, conduct, and successfully terminate, pathological, chemical and hygienic experiments. Again, none with a good practice on his hands has time to devote to these things sufficient to accomplish much, and the dentist who has not a good practice—especially if he has been at work long—would not bring out much by experiments for other reasons. The man of great business and industry, though he might attempt numerous experiments, would find himself interrupted at every turn, and in nine cases out of ten these interruptions would prove fatal to the object, and the experimenter become discouraged, and in future though he thinks of many things he rarely ventures upon an experiment, remembering his former disapointments. Many experiments require long time, and close attention, to secure a happy termination.

The expense in carrying on a series of experiments in the departments named is a heavy item, and all can aid one or two men in doing that much easier than each one can do the whole for himself.

Every one has many experiments that he wishes to see carried out, and he thinks, "as soon as I get time I will." Nine-tenths of these never get out of the brain, where they are conceived, and those that do get out seldom amount to anything.

It is not supposed for a moment, that by the above arrangement every body is going to quit studying. By no means. Every one will do all he can, and be encouraged to do more thinking than before, from the fact that if he has but time to sprinkle his thoughts on paper, and send that to the committee or experimenter their value will be at once thoroughly and efficently tested by careful experiment. In this way, the originator will secure full credit for the suggestions.

This enterprise is wholly at the command of the dental profession of the United States. The person employed will be wholly at your command gentlemen of the profession, at least, so far as you sustain the enterprise, and the result of his labor is your property.

That committee should feel the responsibility of the trust imposed upon them, and select one who is as thoroughly competent, to the trust as possible, and one in whom the profession can repose confidence for the faithful performance of the duty,

We would suggest that the names of all the contributors be published.

This is an enterprise in which we hope the whole profession will take a deep interest, and aid by their contributions and suggestions, equal to the importance of the subject. T.

---

### ADHESIVE FOIL,—BY ARTHUR.

By the politeness of Messrs. Jones, White & McCurdy, we have received a copy of Dr. Arthur's treatise on adhesive foil.

The Dr. has exhibited a perseverance and energy in the pursuance of his favorite subject, that is truly commendable, and in the results obtained has outstriped anything that had been thought of before in regard to foil.

Dr. Arthur does not claim to have done more than some others *could* have done, but he has done what no one did before. In the work before us the Dr. has given his method of working adhesive foil; he describes the instruments and the method of using them; the various kinds and conditions of the adhesive foil, and the manner of using the different varieties. Also the difficulties that present themselves, and the manner of overcoming them. We esteem this as a valuable addition to our library; it is especially valuable because it *is on one* important point, and investigates that thoroughly.

We all like the man of one idea, who can set himself down to one point, for then we expect something will be accomplished, and we are not often disappointed.

This is a work that no operator can afford to do without. It is written in Dr. Arthur's happy style.

It is "*got up*" in Jones, White & McCurdy style. *Nuff ced.*

It can be procured of Jones, White & McCurdy, and of the agents, or those selling their goods.

---

### THE DENTAL SCHOOLS.

Before our next issue the various Dental Colleges will be in full blast. We have already noticed the change in the faculty of the Ohio College. A still greater change has occurred in the Pennsylvania College. Prof. Arthur and Parry are no longer connected with the school, and Prof. Townsend has retired from active duties in it. We have not the pleasure of a personal acquaintance with all of the new incumbents, but we wish them a glorious success. We have not yet seen the announcement of the Baltimore College, and cannot say whether any changes have occurred or not. We hope all three will be well patronized. "We speak that which we do know," when we state that every man who holds a chair in any of the above named faculties does so at a pecuniary sacrifice. It is hoped that this will not always be the case. It should not be, for the best men in the world will tire of any labor, however useful it may be, which is neither appreciated nor paid for.

---

### PROF. SHOTWELL'S PORTRAIT.

For the beautiful and accurate portrait of Prof. Shotwell, which is found in the present number, we are indebted to our friends, the proprietors of the "Cincinnati Medical Observer." This likeness is so accurate, that while gazing on it we fancy we can almost hear the eloquent lectures of our late beloved teacher. We could not think of a neater compliment to our patrons than to give them the portrait of one who was so early and earnestly identified with the cause of dental education in the West. We hope each subscriber will see that it goes down to posterity bound with the present volume of the Register.

---

### REPORTING DISCUSSIONS.

It is always difficult to obtain a *satisfactory* report of the discussions of a public meeting. A verbatim report is generally too voluminous, and, by the way, is not the most likely to satisfy the speakers, as few men like to be reported exactly as they speak. A special difficulty in our profession arises from the fact that professional reporters do not understand our professional terms, and therefore, frequently make mistakes. It is altogether impracticable to submit the report to the revision of all concerned, and even if this could be done, mistakes would occur. As all can not have an opportunity to revise their remarks, it is but fair that none should. For our own part, when we go into a public discussion we make up our mind to be represented correctly, incorrectly, or not at all, as the case may be. We could not ask the prvilege of revising our remarks when we knew others could not have it. It might be very desirable to have them revised, but courtesy to our brethren would require us to forego the pleasure of appearing to better advantage in print than they, when this must be attained by secretly availing ourselves of a privilege which they have no opportunity to enjoy.

---

### AUTHOR OF DENTAL WREATH.

In the list of contributors to the last volume of the Register, for want of the name, we inserted, "Author of Dental Wreath." This occurred immediately after the name of Dr. Chas. H. Roberts, and led at least one person to suppose that it was intended to convey the impression that he was the author of the poem. The period after the doctor's name shows plainly that he is considered one contributor, and the "Author," another. The author of the WREATH is a WOMAN, and her manuscript was by far the neatest that has come into our hands, in our short editorial career. We don't know who she is.

---

### "JOHN PHŒNIX AND THE DENTISTS."

Many of our readers have seen John's account of the late dental excursion to Nahant. We have a right to complain of John. Most people regard his effusions as good natured caricatures, and he should be true to himself in this respect. His account of the valorous "charges" of the profession are in perfect accordance with his general character; but what right has he to approximate the *sober truth*, in the same connection, as he does in the expressions, "every man with a cigar in his mouth." "I observed—two hundred and sixty-eight used tobacco in some form?" John will be delighted to learn that his suggested operation on the *mouth* of the Mississippi is in a fair way to be accomplished next year, as the Convention adjourned to meet on the banks of a noted tributary of that same river.

---

### NEW DENTAL DEPOT.

By reference to our advertising sheet, Dr. Crowell's card will be found. It is not necessary for us to say we will introduce him, for every body knows him.

Dr. Crowell has gone into the manufacture of teeth, and we see by his advertisement he is promising rather more than has been done as yet, by any other, just see his prices of teeth; these teeth, he says, are of the first quality. And even at these prices, he proposes to make a discount on large bills. Now gentlemen of the profession, if you can get such teeth at these prices, "pitch in."

He has on hand, " at lowest prices," all dental materiels and fixtures. I cannot suggest any better method of testing the truth of all the doctor says, than just to try him.

---

☞ By the kindness of our friend John R. McCurdy, one of the editors of the News Letter, we have the proceedings and discussions, of the late American Dental Convention, all in proof, and in very good condition. We will venture the assertion, that Mc put *some* labor upon it. For all this we are very much indebted, and may the time soon come when we can return the favor.

---

### DENTAL DEPOTS.

We must congratulate the profession, of the West, for the very superior facilities which they enjoy for procuring materials and impliments, necessary in the practice of the profession. It would seem a useless work to specify to the profession of the West, what our dental depots have for sale. But, gentlemen, by the way of refreshing your minds, we would just say that you will find at

TOLAND'S,

No. 38 4th street, every thing you ever saw there before, and many of these much better than you ever saw before, for the improvements making on comparatively old things, outstrip all calculation, and you will find a great many new things that you never saw before. Almost every time we step into this establishment, we have to enter into an investigation of some new instrument, appliance, or material. John has been out on an excursion, and we should judge, that nothing had escaped his sharp search. Every new thing that turns up, he seems to have it.

DR. J. M. BROWN,

Corner of 4th and Walnut streets, has a stock inferior to none, his cases and drawers are always filled with the finest selections that can be made anywhere.

The Dr. has been in the trade long enough to know just what you want. He knows just about as well as any of the profession and a great deal better than *some* of them. He knows not only what you want, but he knows where to get it. You will always find at Dr. Brown's a large superior stock to select from.

Every thing can be found in our depots that can be found in the East, and some things that can not be found there. And to the profession of the far west, we would just say, that, DR. A. M. LESLIE, of the St. Louis dental depot, is just as thoroughly prepared, and as ready to supply your requirements, as the most fastidious could wish. The Dr. is a superior practical dentist, of many years successful practice, so in his hands your cases are perfectly safe.

---

### LABIAL FILLING OF UPPER INCISORS.

This is a new, and we think, a very excellent method of filing large cavities upon the anterior surfaces of the front teeth. A full description is given upon another page, taken from the last number of the American Journal of Dental Science. The description is by Dr. Volck, of Baltimore, and the first suggestion from Dr. Maynard. By this method, the very great objection to large brilliant metalic surfaces, upon the front teeth is almost wholly obviated, of course the little metalic ring will be there, but with a good degree of skill this may be made so small as scarcely to be perceptable to the common observer. The method of preparing the operation is so well described that we hardly venture to make a suggestion in regard to it, yet we think it might lessen the difficulty of forming the cavity and the piece, to have about three or four sizes of wheel burs with which to form the cavity, and then a corresponding number of holes in a piece of steel just a little less than the corresponding burs, into which to fit the enamel filling. This would make the operation of fitting very neat and easily performed. There will some times be elliptical cavities that could not be made round, without cutting away too much of the tooth, in such cases the fitting must be done by the eye.

We have just received a beautiful specimen operation of this kind from Dr. Ed. Hale, junior, of St. Louis. This is pearl set in a ring of gold, and looks beautiful, and is very neatly done. Some may object to this operation because it is difficult, but a little practice, with some skill and care will enable any good operator to accomplish it well, but a bungling awkward manipulator should never attempt it. T.

---

### RECEIPTS FOR VOLUME X.

Dr. J. W. Fisher $3 00
Dr. J. A. Brown 3 00
Dr. Wm. H. Smith 3 00
Dr. Wm. M. Hunter 3 00
Dr. W. C. Duncan 3 00
Dr. A. N. Newton 3 00
Dr. J. Holmes 3 00
Dr. N. B. Slayton 3 00
Dr. A. B. Satterthwaite 3 00
Dr. J. L. Walker 3 00
Dr. J. F. Barbour 3 00
Dr. F. Field 3 00
Dr. S. B. Palmer 1 00
Dr. Isaiah Forbs 3 00
Dr. S. P. Miller 3 00
Dr. H. M. Sherrar 3 00
Dr. B. B. Floyd 3 00
Dr. W. D. Jenks 3 00
Dr. S. C. Ingersoll 3 00
Dr. Wm. Howell 3 00
Dr. James Dennis 3 00
Dr. W. S. Chandler 3 00
Dr. Dan'l Daugherty 3 00
Dr. O. P. Laird 3 00
Dr. A. W. French 3 00
Dr. Wm. K. Webster 3 00
Dr. R. G. Hamill 3 00
Dr. G. C. Porter 3 00
Dr. S. C. Gray 3 00
Dr. Purcell 3 00
Dr. G. H. Perrine 3 00
Dr. Dan'l Neal 3 00
Dr. D. S. Levett 3 00
Dr. A. Phillips 3 00
Dr. B. F. Slater 3 00
Dr J. G. Glenn 3 00
Dr. J. Boggs 3 00
Dr. W. M. Rogers 3 00
Dr. J. Murray 3 00
Dr. N. H. Drew 2 00
Dr. J. F. Johnston 3 00
Dr. J. H. Bancroft 3 00
Dr. J. M. Lewis 3 00
Dr. J. C. Hardy 3 00
Dr. Jos. Doughty 3 00
Dr. J. Fertich 1 00
Dr. T. D. Ingersoll 3 00
Dr. J. H. McQuillen 3 00
Dr. J. D. Wingate 3 00
Dr. J. D. Schooley 3 00
Dr. C. T. Cushman 3 00
Dr. J. T. Freeman 3 00
Dr. C. B. Porter 3 00
Dr. J. W. Baxter 3 00
Dr. Aaron Blake 3 00
Drs. Dunham & Hale 3 00
Dr. W. W. Allport 3 00
Dr. H. J. B. M'Kellops 3 00
Dr. Hannaman 3 00
Dr. Sturgiss 3 00
Dr. H. Barron 3 00
Dr. M. S. Jackson 3 00
Dr. W. A. Pease 3 00
Dr. Geo. Porter 2 00
Dr. S. Hinson 3 00
Dr. D. W. Harris 3 00
Dr. G. L. Paine 3 00
Dr. W. A. Chittendon 3 00
Dr. H. H. Harvey 3 00
Dr. Geo. S. Fouke 3 00
Dr. W. Potter 3 00
Dr. E. J. Dunning 3 00
Dr. H. C. Kingsbury 3 00
Dr. J. C. Robbins 3 00
Dr. F. H. Clark 3 00
Dr. B. Lord 3 00
Dr. H. C. Kendrick 3 00
Dr. T. D. Thurman 3 00
Dr. A. Clark 3 00
Dr. Isaac George 1 00
Dr. T. L. Buckingham 3 00
Dr. F. T. Chase 3 00
Dr. Thos. Wade 3 00
Dr. Chas. Goodrich 3 00
Dr. A. H. Moore 3 00
Dr. E. R. E. Carpenter 3 00
Dr. A. W. Sample 3 00
Dr. W. B. Moffatt 3 00
Dr. John Fry 3 00
Dr. Jos. Ramsey 3 00
Dr. Geo. Fredericks 3 00
Dr. E. Osmond 3 00
Dr. I. Hough 3 00

### RECEIPTS FOR VOLUME XI.

Dr. J. Murry $2 00

Dr. W. A. Pease 3 87

Dr. M. Kelly 3 00

Dr. H. A. Smith 3 00

Dr. T. H. Beebe $3 00

Dr. T. D. Thurman 2 00

Dr. S. L, Buckingham 3 00

Dr. Geo. Barrere 3 00

### RECEIPTS FOR VOLUME XII.

Dr. M. Kelly 3 00

